# Going Micro in Leptospirosis Kidney Disease

**DOI:** 10.3390/cells11040698

**Published:** 2022-02-16

**Authors:** Wiwat Chancharoenthana, Asada Leelahavanichkul, Marcus J. Schultz, Arjen M. Dondorp

**Affiliations:** 1 Tropical Nephrology Research Unit, Department of Clinical Tropical Medicine, Faculty of Tropical Medicine, Mahidol University, Bangkok 10400, Thailand; 2 Tropical Immunology and Translational Research Unit, Department of Clinical Tropical Medicine, Faculty of Tropical Medicine, Mahidol University, Bangkok 10400, Thailand; 3 Department of Microbiology, Faculty of Medicine, Chulalongkorn University, Bangkok 10330, Thailand; aleelahavanit@gmail.com; 4 Center of Excellence on Translational Research in Inflammatory and Immunology (CETRII), Department of Microbiology, Faculty of Medicine, Chulalongkorn University, Bangkok 10330, Thailand; 5 Mahidol–Oxford Tropical Medicine Research Unit, Faculty of Tropical Medicine, Mahidol University, Bangkok 10400, Thailand; marcus.j.schultz@gmail.com (M.J.S.); arjen@tropmedres.ac (A.M.D.); 6 Department of Intensive Care, Amsterdam University Medical Centers, 1105 AZ Amsterdam, The Netherlands; 7 Laboratory of Experimental Intensive Care and Anesthesiology (L.E.I.C.A.), Amsterdam University Medical Centers, 1105 AZ Amsterdam, The Netherlands; 8 Centre for Tropical Medicine and Global Health, Nuffield Department of Medicine, Oxford University, Oxford OX3 7LG, UK

**Keywords:** acute kidney injury, immune response, interstitial nephritis, leptospirosis

## Abstract

Leptospirosis is a zoonotic and waterborne disease worldwide. It is a neglected infectious disease caused by *Leptospira* spp., as well as a reemerging disease and global public health problem with respect to morbidity and mortality both in humans and animals. Leptospirosis emerges as a leading cause of acute febrile illness along with hepatorenal injury in many countries, including Thailand. While most affected persons are symptomatic in acute disease, which is always difficult to differentiate from other tropical diseases, there is growing evidence of subtle manifestations that cause unrecognized chronic symptoms. The kidney is one of the common organs affected by Leptospires. Although acute kidney injury in the spectrum of interstitial nephritis is a well-described characteristic in severe leptospirosis, chronic kidney disease from leptospirosis is widely discussed. Early recognition of severe leptospirosis leads to reduce morbidity and mortality. Thus, in this review, we highlight the spectrum of characteristics involved in leptospirosis kidney disease and the use of serologic and molecular methods, as well as the treatments of severe leptospirosis.

## 1. Introduction

Leptospirosis is a zoonotic disease in tropical and subtropical regions. The spread of leptospirosis infection predominantly occurs in the epidemic area during the rainy seasons and flooding. Leptospirosis remains a huge global public health problem with increasing prevalence due to global warming and climate change. Outdoor activities, such as rafting, canoeing, triathlons, or bathing in natural bodies of water, have been reported as risk factors for leptospirosis infection [1,2], while occupational risks are associated with some careers, including farm work, veterinary medicine, military, field-work research, and living near a rubber tree plantation [2,3]. The disease transmission of leptospirosis mostly occurs from direct contact (especially with the non-intact skin barrier of the host) with contagious secretions (urine) from the infected or carrier animals as well as environmental contaminants, such as water and soil, where pathogenic *Leptospira* can survive for several weeks [2]. Several wild animals have been reported as reservoir hosts, but the brown rat (*Rattus norvegicus*) is the most important reservoir reported in many regions worldwide [4]. Despite the inconclusive pathogenesis of leptospirosis, the interplay between host immune responses and the spirochete (*Leptospira* strain) is recognized as critical for the disease presentation in the acute or chronic phases.

The kidney is the main target of *Leptospira* in both the acute and chronic phases of infection. In fact, the clinical course of severe leptospirosis is biphasic characteristic [1,2,3,4]. First, leptospires penetrate the host mucocutaneous barriers leading to an immune phase that causes overwhelming antibody production as well as urinary shedding. Then, in the immune phase, leptospires are eliminated from systemic organs but not kidneys [2]. Hence, the distribution of leptospires in the kidney during the acute phase may affect the deterioration of renal function during the chronic phase. Acute kidney injury (AKI) following leptospirosis is characterized by acute tubulointerstitial nephritis from either immune response against microbial molecules or other factors (such as hyperbilirubinemia or rhabdomyolysis-induced myoglobinemia), accompanied by renal tubular micro-obstruction. The subacute and chronic presentation of leptospires in renal proximal tubules in a carrier state can progress to chronic tubulointerstitial nephritis (CTIN) and fibrosis [5]. Consequently, the manipulation of host immune responses to the pathogen might prevent chronic kidney disease (CKD) and multi-organ damage from leptospirosis. For this reason, asymptomatic leptospirosis kidney disease should be considered for the differential diagnosis of renal fibrosis and CKD of unknown causes, particularly for patients who live in epidemic areas.

The diagnosis of leptospirosis is also a key step toward a better outcome. Although several rapid and improved diagnostic tests for leptospirosis are now available [6], negative results should not be considered to exclude leptospirosis, especially in highly suspicious cases. Empirical therapy should be started concurrently with suspicion of a diagnosis of leptospirosis because early antimicrobial therapy administration may prevent patients from progressing to more severe forms of the disease.

## 2. Epidemiology

**Figure 1:**
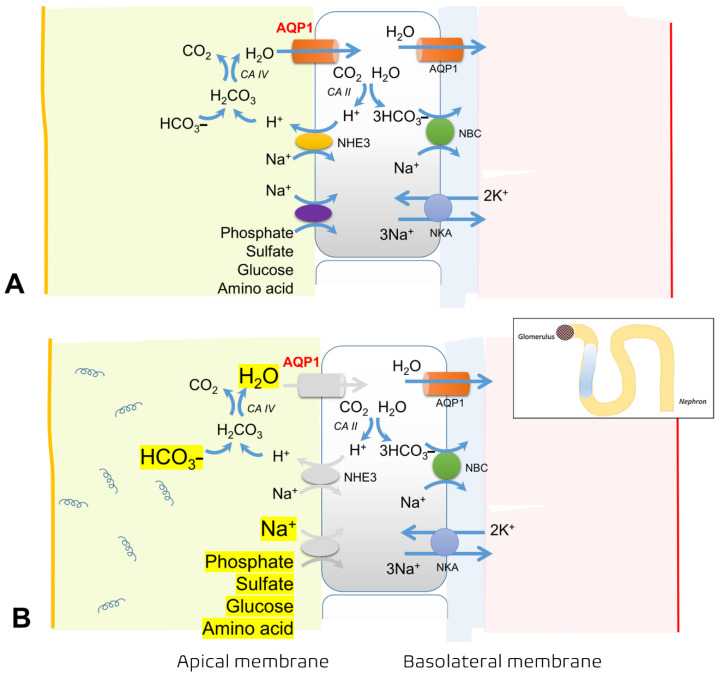
The mechanisms of proximal tubular defects caused by *Leptospira* spp. (**A**) Illustration of normal physiology and the important channels involved in the regulation of intraluminal bicarbonate, phosphate, sulfate, glucose, and amino acids via the aquaporin (AQP)-1 channel, sodium–hydrogen exchanger (NHE) 3 (or sodium–hydrogen antiporter 3), and sodium/phosphate cotransporter (Na/Pi). (**B**) Leptospira causes injury along proximal tubules, leading to altered regulation of these luminal gate channels in both apical and basolateral membranes, for instance, the reduction in NHE3, AQP1 channels, and the decreased expression of α-Na^+^/K^+^-ATPase along the apical and basolateral membranes, respectively. Hence, in the luminal part, there is an accumulation of free water (causing polyuria), sodium wasting, and characteristics of Fanconi’s tubular dysfunction, including bicarbonaturia, hyperphosphaturia, and glucosuria.

Although the actual incidence of leptospirosis worldwide is not precisely known, the age and gender-adjusted disease morbidity models estimated annually 1.03 million cases and 58,900 deaths worldwide [7], where the highest estimated of disease morbidity and mortality were observed in South and Southeast Asia, Oceania, Caribbean, Andean, Central, and Tropical Latin America, and East Sub-Saharan Africa [7]. In Thailand, the incidence of leptospirosis is reported at around 6.6 per 100,000 population, with a 1.5% fatality rate. The northeastern region has the highest incidence (12.5 per 100,000 population), depending on the season, and the highest incidence occurs during the rainy season from August to October each year [8]. According to a recent study by Torgerson et al. [9], a total of 2.90 million Disability Adjusted Life Years, which estimated >70% of the global cholera burden as the estimation of the 2010 Global Burden of Disease (GBD) reports [10]. As such, leptospirosis seems to be the biggest zoonotic-related health disease burden [7]. Furthermore, with the increasing frequency of flooding due to global warming in both developed and developing countries, leptospirosis is not limited to tropical countries. Another aspect of the problem is a semantic or epistemological issue because over 50% of leptospirosis patients with or without kidney involvement are asymptomatic or exhibit mild symptoms [11]. Thus, it is important to consider the rates of leptospirosis in patients with infections with AKI involvement or those with CKD of uncertain etiology, as leptospirosis could contribute to the unrecognized pathogenesis. The complications of leptospirosis can include multiple organ damage in 5–10% of total infected cases [12]. Moreover, leptospirosis causes AKI, at rates varying from 10–88% depending on the definition of AKI [13,14]. The Kidney Disease Improving Global Outcomes (KDIGO) clinical practice guidelines for AKI in 2012 define AKI as a change of either serum creatinine (SCr) exceeding 0.3 mg/dL within 48 h or an increase in SCr to 1.5 times the baseline value within the previous one week along with decreased urine output (Figure 1). In Teles et al.’s [14] retrospective study of 205 leptospirosis patients with AKI, 55.1% of patients with AKI were classified as KDIGO 3, while 16.1% were classified as KDIGO 1 and 17.6% as KDIGO 2. However, the observed data seem to indicate a high prevalence of AKI as defined by the KDIGO criteria despite the mild expression of leptospirosis. This could be due to oversensitivity of the KDIGO criteria, or a high incidence of AKI in leptospirosis regardless of the degree of severity. Robust validation of the true incidence of AKI in leptospirosis with diverse definitions is needed. The risk factors of leptospirosis vary based on the country and area of study and can be classified as occupational, behavioral, and environmental risk factors. Kamath et al. [15] conducted a population-based case-control study in Southern India in which occupational factors, such as outdoor activities (odds ratio [OR] of 3.95) or the presence of cuts or wounds on body parts during work (OR: 4.88), and environmental factors, such as contact with rodents through food contamination (OR: 4.29) or contact with soil or water contaminated with rat urine (OR: 4.58), were found to be associated with leptospirosis. Meanwhile, a retrospective study from Thailand revealed that living near rubber tree plantations as well as bathing in natural bodies of water two weeks before the illness was significantly associated with an increased risk of severe leptospirosis compared to non-severe leptospirosis (OR: 12.00 and 7.25, respectively). Notably, most patients were exposed while bathing in stagnant water (41.9%), slowly flowing water (29.0%), and mud (29.0%) [2]. Moreover, leptospirosis cases are increasing in terms of travel-related infection. The estimated annual incidence of travel-related leptospirosis in the South-East Asia region is approximately 1.78 per 100,000 travelers per year compared with an incidence of endemic cases of 0.06 per 100,000 population per year (risk ratio [RR] 29.6), which are predominately related to water-related activities [16].

## 3. Pathophysiology

### 3.1. Acute Kidney Injury in Leptospirosis

AKI is a common manifestation of leptospirosis. Renal involvement in leptospirosis varies from asymptomatic urinary abnormalities to severe AKI that requires supportive dialysis. The most common renal pathology in leptospirosis is acute tubulointerstitial nephritis (ATIN), whereas hypokalemia and sodium wasting are the common laboratory findings. Interestingly, AKI caused by leptospirosis is usually non-oliguric, and hypokalemia accounts for 45–50% of all AKI cases [17]. ATIN from leptospirosis is characterized by diffuse interstitial edema and mononuclear cell infiltration. Notably, glomerular involvement in leptospirosis is less common. Leptospirosis-induced vasculitis is rare, with unfavorable renal outcomes that can be attenuated by corticosteroid administration [18,19,20,21]. Different mechanisms have been proposed for non-oliguric, hypokalemic AKI in leptospirosis (discussed below).

#### Tubular Dysfunction and Related Electrolyte Disturbances

Several tubular defects have also been reported, such as bicarbonaturia, glucosuria, decreased proximal tubule sodium reabsorption, and high excretion of phosphate and uric acid, also known as Fanconi syndrome [11,22,23]. Reductions in sodium–hydrogen exchanger isoform 3 (NHE3), which is expressed along with aquaporin 1 (AQP1) in the apical membrane of the proximal tubule, and decrease in α-Na^+^/K^+^–ATPase [24] cause several complications. Hyponatremia in leptospirosis is attributed to several causes, including increased urinary sodium loss, cellular efflux of sodium from Na^+^/K^+^–ATPase defects, increased levels of antidiuretic hormone (ADH), and resetting of the osmoreceptors [25]. Accordingly, the combination of the clinical clues of hyponatremia, hypokalemia, and non-oliguric AKI (or polyuria) are unique characteristics of leptospirosis nephropathy.

**Figure 2:**
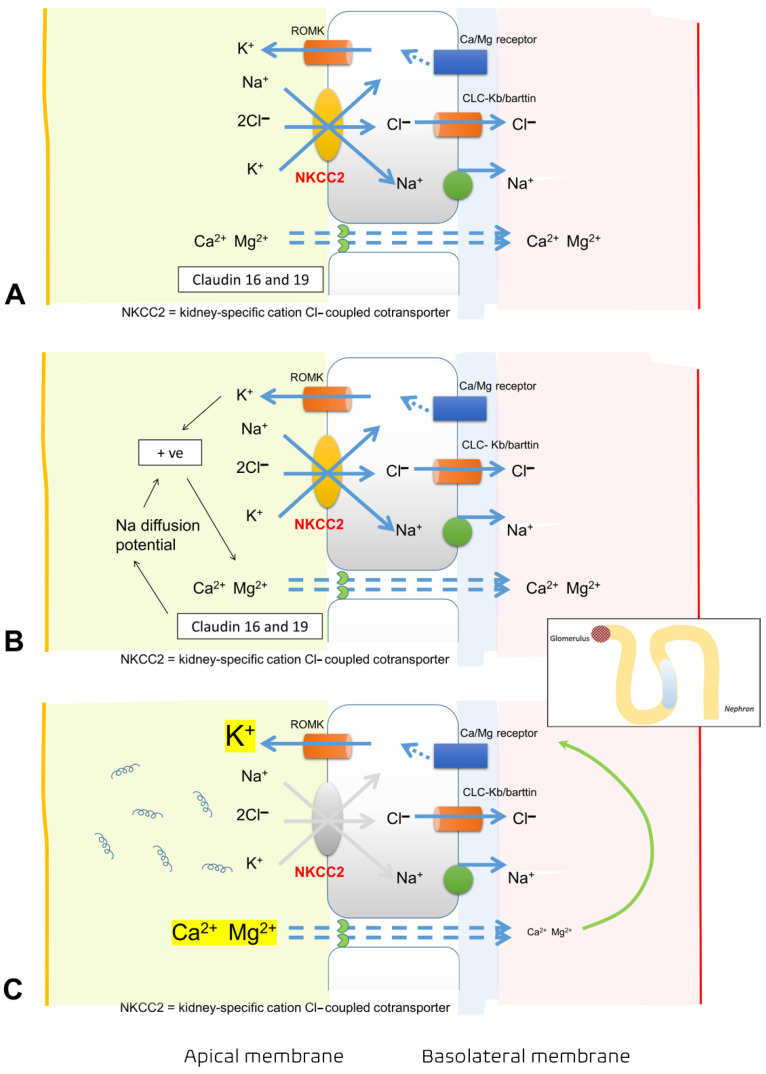
Illustration of the pathogenesis of hypokalemia, hypomagnesemia, and hypocalcemia in leptospirosis kidney disease. (**A**) Sodium-potassium-2 chloride cotransporter (kidney-specific cation Cl- coupled cotransporter, NKCC2) is an important cotransporter that maintains the homeostasis of intraluminal cations and anions. (**B**) NKCC2 functions with potassium channels (ROMK) and paracellular selective protein channels of magnesium (main) and calcium (minor), called claudin 16 and 19, in order to maintain intraluminal positive electric charge. (**C**) Similar to pharmacological inhibition of NKCC2 by furosemide, loss of NKCC2 controlling due to tubular injury caused by *Leptospira*, leading to loss of intraluminal positive electric charge, which causes decreased reabsorption of magnesium and calcium. Thus, low levels of both magnesium (hypomagnesemia) and calcium (hypocalcemia) in circulation, in turn, provide positive feedback (green arrow) and enhance potassium excretion into the lumen to maintain homeostasis. In fact, the Ca/Mg receptor is located at the basolateral membrane so far called calcium sensing receptor (CaSR), which is the key molecular player involved in sodium, potassium, and chloride transport by the thick ascending limb. During hypocalcemia and/ or hypomagnesemia, inactivation of basolateral CaSR enhances ROMK. Eventually, a large amount of potassium will be lost in urine.

In addition, downregulation of the sodium-potassium-2-chloride co-transporter (NKCC2) in the medullary thick ascending limb (mTAL) of the loop of Henle may also explain the loss of sodium and potassium in the urine [24,26,27] (Figure 2). Polyuria or non-oliguric AKI occurring during the first stage of leptospirosis might be another symptom related to the reduced expression of aquaporin 2 (AQP2) and a urinary concentration defect due to resistance of the inner medullary collecting duct to vasopressin. During the recovery phase of AKI, AQP2 expression increases as a compensatory mechanism [24].

Hypomagnesemia is also common in leptospirosis patients with AKI from magnesium wasting [28]. One experimental study also reported renal magnesium wasting secondary to decreased NKCC2 on the apical membrane of mTAL [27] (Figure 2). Accordingly, hypomagnesemia and hypophosphatemia caused by hypermagnesuria and hyperphosphaturia, respectively, should be closely monitored during the course of leptospirosis infection. Hypomagnesemia from tubular dysfunction in leptospirosis may cause dramatic changes in magnesium homeostasis, which needs substantial amounts of magnesium replacement, especially in patients with myalgia, lethargy, and arrhythmias. On the other hand, rapid correction of hypomagnesemia may cause unintentionally increase circulating magnesium levels because leptospirosis may contribute to AKI, which may decrease magnesium clearance. Likewise, severe hypophosphatemia (defined as serum phosphate <1.5 mg/dL) from proximal tubulopathy may contribute to metabolic encephalopathy and myopathy. Accordingly, as the authors’ experience, serum magnesium and phosphate levels should be evaluated every 3–5 days and 1–2 days, respectively, particularly in severe leptospirosis.

Interestingly, leptospirosis-related AKI occasionally requires supportive renal replacement therapy in the acute phase of infection. The kidneys may fully recover after the complete course of early antimicrobial therapy. Effective treatment of leptospirosis reversed tubular dysfunction in an in vitro study [27]. Leptospirosis patients also have more favorable outcomes with non-oliguric than with oliguric AKI [17].

### 3.2. Leptospirosis with Systemic Inflammatory Response Syndrome (SIRS)

Severe leptospirosis is complicated by sepsis and septic shock [29,30]. In the early phase, leptospirosis is related to an overwhelming activation of inflammasomes and proinflammatory cytokines, causing kidney inflammation and subsequent damage. Leptospira can be found in the proximal tubular cells at day 10 of the infection, and it can subsequently be found in the tubular lumen at day 14 of the infection [31]. Its antigens are also found in the proximal tubular cells, macrophages, and the interstitium [32].

The outer membrane proteins (OMPs) of *Leptospira* contain antigenic and virulent compounds, including lipoproteins, lipopolysaccharides (LPS), and peptidoglycans, which determine the host responses. In animal models of sepsis, LPS or endotoxin cause detrimental effects to the host [33,34]. Leptospira LPS, located on the OMP, appear to be the major antigens that affect immunity to *Leptospira* and believe its functions relevant to host–pathogen interactions which determine virulence and pathogenesis. To elucidate the mechanisms of tubule-interstitial injury caused by *Leptospira*, the *Leptospira*l OMPs were extracted on cultured mouse renal epithelial cells, which showed the expression of a variety of genes related to tubular cell injury and inflammation [35]. The *Leptospira*l OMPs activate nuclear transcription factor kappa B (NF-KB), activator protein-1, and several downstream genes expressed in the medullary thick ascending limb cells [35]. LipL32, a major pathogenic lipoprotein on the OMP, induces tubulointerstitial nephritis-mediated gene expression in mouse proximal tubular cells and is a prominent immunogen during leptospirosis infection in humans [36]. In addition, LipL32 is a hemolysin that causes hemolysis of erythrocytes during *Leptospira* infection [37], and it directly affects proximal tubular cells by substantially increasing the gene and protein expression of several pro-inflammatory cytokines, including inducible nitric oxide (iNOS), monocyte chemoattractant protein-1 (MCP-1), and tumor necrosis factor-α (TNF-α). Therefore, the identification of novel OMPs of the *Leptospira* should remain a primary focus for increasing knowledge of leptospirosis pathogenesis and treatment.

Toll-like receptors (TLRs) are proteins that recognize specific molecular patterns of pathogens and represent the first line of immune defense mechanisms in the innate immune response. The effects of TLRs were evaluated to determine whether TLRs could mediate the inflammatory response induced by *Leptospira*l OMP in renal proximal tubular cells. Interestingly, only TLR2 but not TLR4 increased the expression of iNOS and MCP-1. Accordingly, the findings indicate that the stimulation of iNOS and MCP-1 caused by pathogenic *Leptospira*l OMPs, in particular LipL32, in proximal tubular cells requires TLR2 (usually co-expressed with TLR1) for the early inflammatory response [5].

Then, a cascade of inflammation is activated in the renal tubular cells, as leptospirosis induces interleukin (IL)-1β and IL-18 secretion from human macrophage cells through reactive oxygen species and cathepsin B mediated-NLRP3 inflammasome activation [38]. Other circulatory cytokines and chemokines, including IL-6, IL-10, monocyte chemoattractant protein-1 (MCP-1), and TNF-α [39], are also produced during leptospirosis infection. Acute cytokine and chemokine surges occurring in leptospirosis patients can cause a detrimental syndrome of sepsis and severe sepsis due to an imbalance between pro- and anti-inflammatory responses. Increased levels of MCP-1, IL-11, and small inducible cytokine A2 occur during leptospirosis with thrombocytopenia [40]. These findings infer a role of cytokine and chemokine production during the acute and subacute phases of leptospirosis infection. Conversely, chronic inflammasome activation may be the pathway that leads to renal parenchyma fibrosis or CKD [41].

AKI following leptospirosis may thus arise due to acute tubular necrosis (ATN) from ischemia and poor renal tissue perfusion as the result of sepsis and septic shock. In addition, sepsis-associated AKI (sepsis-AKI) in leptospirosis is also possible, especially in patients with a low blood pressure episode. Evidence from both experimental and clinical studies shows that septic shock develops into a sepsis-related immunosuppression state, leading to the death of the host because of innate and adaptive immunity disturbances [42]. Therefore, circulating cytokines and chemokines may interfere with the immune response in severe leptospirosis; for example, low circulating neutrophils levels may be associated with impaired antimicrobial activity [43,44,45].

### 3.3. Chronic Kidney Disease in Leptospirosis

The consequences of ATIN caused by leptospirosis infection include tubular atrophy and interstitial nephritis in the event of unsuccessful treatment or incomplete recovery. In an attempt to elucidate the causal association between *Leptospira* and renal fibrosis, the effects of OMP from pathogenic *Leptospira* on the production and accumulation of extracellular matrix have been explored [46]. The binding of *Leptospira*l OMP to proximal tubular cells, HK-2 cells, led to an increase of type I and type IV collagens in a dose-dependent manner. Likewise, the active transforming growth factor (TGF)-β1 secretion was increased twice following the addition of *Leptospira* OMP, while anti-TGF-β1-neutralizing antibodies attenuated the increased production of type I and type IV collagen, indicating the participation of TGF-β1 in the cascade. This phenomenon was confirmed by the increased nuclear translocation of SMAD3 after the administration of *Leptospira*l OMP, and overexpression of the dominant-negative SMAD3 prevented the *Leptospira*l OMP-induced increase of type I and IV collagen production without any effects on metalloproteinase activity [46]. This clearly demonstrated the effects of *Leptospira*l OMP in terms of enhancing extracellular matrix synthesis mediated by the TGF-β1/SMAD pathway.

Although data from both in vitro and in vivo studies indicate the possibility of CKD and renal fibrosis due to leptospirosis infection [46,47,48], the results from both a meta-analysis [49] and a long-term, three-year follow up study are inconsistent, as no leptospirosis patients with dialysis dependence were reported [50]. Chronic tubulointerstitial nephritis (CTIN) is a common lesion associated with long-standing leptospirosis that may lead to CKD of unknown etiology and subsequent renal failure. Sustained tubulointerstitial lymphocyte infiltration might be a key factor resulting in the progression to CKD [50]. Perhaps, TLRs may be involved in the AKI–CKD continuum in leptospirosis because TLRs are known to be a key factor in the primary response to both innate and adaptive immunity [47] as well as ischemia-reperfusion injury, glomerulonephritis, and sepsis-AKI. Therefore, further studies should seek to identify the molecular factors that may act as a danger signal by triggering the inflammatory response to different exogenous and endogenous noxious stimuli.

## 4. Host Immunity Responses to *Leptospira* spp.

The main route of *Leptospira* is direct penetration of mucous membranes or opened skin through blood, tissues, urine, or contaminated water, which spread throughout the systemic circulation and disseminate into several organs, such as the liver, lung, and proximal tubule of the nephron in kidneys, where it can multiply and survive for a month [51]. In the initial phase of infection, the innate immune response plays a fundamental role as the first line of defense in leptospirosis, affecting the response of pattern recognition receptors, macrophage phagocytosis, induction of extracellular neutrophil accumulation, and intervention of the complement system [52].

The immune response in humans plays an essential role in the development of clinical signs, as do the virulence factors of some pathogenic strains. Most human leptospirosis is asymptomatic or mild, and only 10% of cases develop severe forms with multiorgan failure and increased mortality rates related to a cytokine storm and an immuno-paralysis status. Pathogenic species stimulate a humoral response in the bacteremia phase, although the subsequent behavior of the immune response is unclear. In studies of patients with multiple clinical manifestations with leptospirosis, the severity of the clinical picture has been associated with various serum cytokines levels, such as TNF-α, IL-10, and IL-6 [53], but this conclusion remains controversial [54]. These conditions have led to increased interest in understanding the immune response in general and the biological events that occur during infection.

### 4.1. Leptospira and the Complement System

The proteolytic activity of *Leptospira*l proteases against the extracellular matrix allows the infection to enter the circulatory stream. The complement system in the circulatory system is one of the main mechanisms of the innate immune response to pathogenic *Leptospira*. However, *Leptospira* has developed strategies to escape the attack of the complement system by binding proteins to complement molecules and producing proteases, such as thermolysin, a thermostable neutral metalloproteinase enzyme that affects the C3 protein chain. Pathogenic strains bind factor H (FH), complement factor H-related protein 1 (FHR-1), factor-H-like protein 1 (FHL-1), and C4b binding protein (C4BP), all of which are responsible for inhibiting the complement system [55]. There are three main strategies for complement evasion of pathogenic *Leptospira* [55,56] through the interference on host complement regulators, host protease, and bacterial protease. For complement regulators, *Leptospira* uses Factor H (FH; a regulator of alternative complement pathway) and C4 binding protein (C4BP; a regulator of classical and lectin complement pathways) to accelerate the decay of the C3 convertases and also act as cofactors for Factor I for the cleavage of C3b and C4b [57,58]. Leptospiral protein (LcpA), together with vitronectin (Vn; a terminal pathway regulator) binds C9 and inhibits its polymerization [59], thus potentially blocking membrane-activating complex (MAC) formation [60]. Additionally, pathogenic *Leptospira* binds several host proteases, such as Urokinase-type plasminogen activator (uPA), that induce active plasmin [61] (a serine protease) for digestion of C3b, C4b, and C5 on the *Leptospira* surface [62]. Moreover, metalloproteases and thermolysin, the endogenous *Leptospira* protease, are able to cleave and inactivate several complement proteins (C2, C3, C4, and Factor B) [60]. The combination of complement regulators, from the host and pathogenic *Leptospira*, facilitates bacterial colonization in the target organs of the host [63].

### 4.2. Leptospira and Neutrophils

During infection, the pathogenic species of *Leptospira* trigger strong activation of neutrophils and a pro-inflammatory response. Neutrophils are important to microorganism control, but they also contribute to tissue damage in the host. It has been demonstrated that low levels of extracellular neutrophil accumulation due to low neutrophil activity in the circulation increase leptospiremia, indicating that the intravascular formation of neutrophil recruitments may be critical in preventing the early spread of pathogenic *Leptospira* [64].

### 4.3. Leptospira and Macrophages

In the initial phase of *Leptospira* infection, macrophages play an important role in the elimination of the spirochete. This effector mechanism together with an efficient cytokine response is related to phagocytic control and the spread of infection, although *Leptospira* spp. has been shown to induce apoptosis in macrophages via the Fas/FasL-caspase-8/-3 pathway and facilitate their survival and proliferation in the host due to the release of mitochondrial factor-inducing apoptosis and endonuclease in infected macrophages [65]. Recent research has been conducted on the micro-transcriptome of macrophages infected with *Leptospira* to determine the epigenetic mechanisms, such as post-transcriptional regulation by RNA. Macrophages infected with *Leptospira* have shown that microRNAs (miRNAs) are regulated by infection with the spirochete, demonstrating that this regulation is vitally important in the response of these immune system cells in *Leptospira* infection [66]. In human macrophages, the activation of the inflammation mediated by reactive oxygen species and lysosomal cathepsin B generated by *Leptospira* infection has prominent levels of NLRP3 and IL-18, favoring the production of IL-1 and leading to a high possibility of phagocytosis and the rapid modeling of some cytokines correlated with leptospirosis resistance [67].

### 4.4. Humoral Response to Leptospira

The specific humoral response against *Leptospira* is characterized by the production of IgM and IgG antibodies on the third day of symptom onset. In addition, IgA production has been reported from day one to the ninth post-infection month; in fact, the increase in IgM has been used as an alternative measure in the early diagnosis of leptospirosis [68]. The effect of the humoral response in humans with leptospirosis allows the serological diagnosis to be made after natural infection [69]. The most important aspect of the humoral response in *Leptospira* infection may be the formation of protective antibodies. Vaccines currently available for leptospirosis are composed of complete cell preparations and have limitations, such as low efficacy, multiple side effects, low immune memory generation, and a lack of cross-protection for serovar differences. Thus, the development of new vaccines is necessary for disease control, and research is currently being conducted to produce vaccines from genetic immunization [70]. *Leptospira’s* OMPs have been considered an antigen, with better immunogenicity for B lymphocytes, which include the structural epitopes OmpL187-98, OmpL1173-191, OmpL1297-320, LipL4130-48, LipL41233-256, and LipL41263-282 [71]. These epitopes are promising candidates for the development of a universal vaccine.

### 4.5. Adaptive Immune Response to Leptospira: T-Cells and Cytokines

Pathogenic species of *Leptospira* induce an immune response mediated by T and B lymphocytes, accompanied by the expression of cytokines that may be associated with gravity charts. Activation of T cells during infection with *Leptospira* initiates the inflammatory response, mainly through cytokine production. This is critical in the early elimination of infection, but the uncontrolled production of pro-inflammatory cytokines can also result in cytokine overproduction followed by a state of immuno-paralysis, which can lead to sepsis and multiorgan failure. T lymphocytes produce IL-1, IL-6, IL-12, interferon (IFN)-γ, and TNF-α, which function as chemo-attractants to recruit leukocytes at the site of infection. Interestingly, in ex vivo, the naïve γδ T cells proliferated in cultures of peripheral blood mononuclear cells (PBMCs) stimulated with antigens of *Leptospira* along with produced both IL-17 and IFNγ [72]. Moreover, whereas leptospirosis-infected wild-type mice demonstrated higher levels of plenty of cytokines, including IL-4, IL-10, and IL-13 compared with uninfected mice, *Leptospira*-induced murine chronic nephritis (Daf1-/- mice) showed higher expression of smooth muscle actin, IL-10, IL-13, but no difference of IL-12 and IL-17 levels [73]. As such, Daf1 and IL-17 may play a crucial protective role in CKD progression as the link between *Leptospira*-induced murine CKD with renal fibrosis [72,73]. However, the hypothesis of selected cytokine response in disease progression is still inconclusive in human leptospirosis so far. In patients with severe leptospirosis and pulmonary involvement, elevated levels of IL-6, CXCL8, and IL-10 were obtained when compared to patients with mild leptospirosis [53]. These high concentrations of cytokines in patients with leptospirosis highlight their key role in the development of severe leptospirosis.

In patients with severe infection, CD4+ T lymphocytes with a pro-inflammatory profile and producers of IL-2 and IFN-γ are found while CD4+ CD25^high^ producers of IL-10 are almost absent. The regulation of T lymphocyte-mediated immune response does not appear to prevent tissue damage generated by the inflammatory response to *Leptospira.* In CD4+ T lymphocytes of humans with severe leptospirosis, the production of TNF-α and other pro-inflammatory cytokines has been found to be higher than in those with the mild form of the disease, which indicates that these cytokines can be used as markers of severity in the immunological phase of infection [74]. The production of IL-10 has been associated with the risk of death in humans with leptospirosis; however, a study conducted on exposed and asymptomatic humans associated the production of this cytokine with the control of the inflammatory response and survival [74]. During *Leptospira* infection, inflammatory mediators and the action of some leukocyte cells, such as T lymphocytes, are quickly regulated in resistant individuals, in contrast to what occurs in individuals with severe forms of leptospirosis [75].

To sum up, the immune response to *Leptospira* infection is not fully understood due to the clinical behaviors of patients with leptospirosis, who interact with the different virulence factors of the bacterial genome, which give *Leptospira* a solid pathogenic capacity.

## 5. Clinical Manifestations

The clinical manifestations of leptospirosis are diverse, ranging from mild, non-specific symptoms, such as flu-like symptoms (fever, myalgia, and headache), to severe symptoms along with end-stage organ injuries (e.g., AKI, acute hepatic failure, and bleeding diathesis, also known as Weil’s syndrome), acute hemoptysis from pulmonary hemorrhage, acute confusion from aseptic meningoencephalitis, and acute heart failure from acute or subacute myocarditis [7].

Accordingly, the three indicators of suspected leptospirosis infection consist of (i) acute febrile illness, (ii) jaundice, and (iii) acute kidney injury [12]. The predictors of a severe form of leptospirosis are severe myalgia at onset, severe bleeding tendency, and marked jaundice. Interestingly, the presence of either pre-existing chronic kidney disease (small kidney size) or enlarged, congested kidneys with anuria is related to the worst outcome [12]. Thus, patients with leptospirosis-associated renal disease should be treated promptly.

## 6. Diagnosis

### 6.1. Clinical Diagnosis

A diagnosis of leptospirosis is based on the history of exposure, risk factors, and clinical manifestations. A high index of suspicion can circumvent later organ damage. However, symptoms of leptospirosis are often mistaken for other causes of acute febrile syndrome, such as dengue infection, malaria, hepatitis, and active autoimmune disease. The lack of pathognomonic signs of leptospirosis means that the diagnosis is tentatively based on the evaluation of fever and myalgia in patients from an endemic area. For this reason, laboratory diagnosis of leptospirosis is essential.

### 6.2. Laboratory Diagnosis

Leptospirosis is difficult to diagnose in the laboratory, especially during the acute phase. It can be performed by directly identifying spirochetes or their components in bodily fluids or tissues, isolating leptospires in culture, or detecting particular antibodies throughout different clinical phases. Leptospirosis is difficult to distinguish from illnesses, such as malaria, dengue fever, rickettsia, influenza, hepatitis, and yellow fever because of its vague clinical appearance. As a result, lab tests are needed to confirm the diagnosis. The detection of antibodies against leptospires, leptospires themselves, or their deoxyribonucleic acid is the basis for these tests (DNA). Current laboratory diagnosis includes both direct identification (the detection of *Leptospira* spirochetes or DNA in the samples or isolation of the organism from specimens) and indirect detection (serological diagnosis or serology for identifying *Leptospira*l infection, which is based on the detection of specific antibodies against various *Leptospira*l antigens) (Table 1).

#### 6.2.1. Direct Microscopic Examination, Culture, and Antigen Detection

Leptospires are thin, bright, actively motile spirals with characteristically rapid spinning (twitching motility) under the conventional darkfield microscope (DFM) and approximately, 10 leptospires/ mL are necessary for the detection of one cell per field. With one week longer in the duration of the infection, there is approximately 10% deceased in yield of DFM (from 100 to 90%). To enhance the sensitivity, several special staining methods; Warthin–Starry silver staining [76] and immunostaining (immunohistochemistry, immunofluorescence, immunomagnetic antigen capture, and immunoperoxidase staining [77]) that requires the serovar specific primary antibodies (in isolation or in combination).

In parallel, *Leptospira* culture from blood, urine, cerebrospinal fluid, and biopsy tissue is possible during the first few days to 10 days post-onset of symptoms of illness (leptospiremic stage). Because they are slow-growing (6–8 h doubling time), fastidious and prone to contamination, the culture samples must be kept for at least 3–4 months before being discarded as negative) with only 23% sensitivity but it is necessary for the drug sensitivity test [6]. As such, EMJH (Ellinghausen–McCullough–Johnson–Harris) media (oleic albumin complex) consists of bovine serum albumin (fraction V), Tween 80, ammonium chloride thiamine, monopotassium phosphate, disodium phosphate, and various nutrients [78] is most commonly used, while the more specialized T80/40/LH media (polysorbate 40, lactalbumin hydrolysate, superoxide dismutase, and rabbit serum), is required for some serovars [79]. For the primary isolation of the large and diverse range of fastidious pathogenic leptospires, Hornsby–Alt–Nally (HAN), seems to be a good media [80].

Detection of *Leptospira*l antigens by immunoperoxidase, immunofluorescence, or an immunomagnetic antigen-capture system has been developed for specimens with low bacterial burdens or dark-field microscopy cannot be used but is not routinely performed due to the limitation on primary antibody [77]. In contrast, nucleic acid recognition with novel DNA amplification; polymerase chain reaction (PCR) (i.e., nested-PCR, quantitative PCR [qPCR]), loop-mediated isothermal amplification (LAMP), and next-generation sequencing (NGS) is valuable for an early and accurate laboratory diagnosis, especially with the *Leptospira* isolation using biological media inoculation and DNA hybridization (DNA probe) [81,82].

A variety of *Leptospira*l targets (such as 16S ribosomal RNA) or DNA can be amplified for diagnosis [83] and real-time PCR (RT-PCR) is more sensitive and specific than standard PCR [84]. Because some RT-PCR primers may bind to a non-specific site, leading to false positive results, most recent real-time multiplex PCR assays have been developed using two sets of primers [85]. Nested PCR also helps in detecting more specific and sensitive DNA sites with additional sets of primers [86,87]. However, current trends for leptospirosis diagnosis are the use of both serological and molecular techniques, such as PCR and ELISA (easier than the gold standard serological MAT), for resource-limited countries [88,89]. Additionally, the LAMP technique [90] to detect a 16S rRNA gene (rrs), is a cost-effective, rapid and high-yield for detecting the pathogenic leptospires in the urine. Currently, NGS is the most precisely-based culture-independent method on core genome analysis in body fluids (blood and urine) [91] and the future direction of leptospirosis tests would be to move towards the molecular classification of leptospires, which overcomes the limitation of culture isolation of leptospires from clinical samples. Hence, the value of PCR in the clinical diagnosis of leptospirosis is particularly good and several modern techniques are emerging.

#### 6.2.2. Anti-Leptospira Antibody Detection

Several serological diagnoses are used; for example, genus-specific antibody tests (indirect hemagglutination [IHA], enzyme-linked immunosorbent assay [ELISA], Lepto Dri Dot [latex agglutination], microcapsule agglutination [MCAT]), and serovar-specific antibody tests (microscopic agglutination test [MAT]) [92,93]. As such, MAT criteria for diagnosis are (i) a fourfold titer rise in paired sera (seroconversion of the current infection) or (ii) a single high titer (≥1:400 or 1:800, higher in epidemic areas) (seropositivity) (antibodies may persist for some time after infection or the cross-react with other diseases). Additionally, MAT requires a live panel of all *Leptospira* serovars in the region, with a panel of locally standardized serovars [93,94] following the World Organization for Animal Health (19 antigens representative of 15 serogroups) [95]. Because antibodies are detectable at 5–7 days post-infection, the MAT test can be negative (titer < 1:50) during the first few days of infection. Diagnosis of leptospirosis can be performed by urine samples [96] using both serology and molecular detection (16S rRNA), particularly in patients with recent infection (MAT ≥1∶800 or ELISA IgM-positive or both) [97].

With limited resources, a clinical prediction score [98] based on the relevant clinical history and related laboratory tests with scoring in seven aspects is as follows: hypotension (3), jaundice (2), muscle pain (2), AKI (1.5), low hemoglobin (3), hypokalemia with hyponatremia (3), and neutrophilia (1) is proposed (a cutoff summarized score of 4 has the area under the receiver operating characteristic curve 0.78 (95% CI 0.68–0.89) for leptospirosis diagnosis [77]). This Thai-Lepto-on-admission probability score could be a diagnostic tool for early presumptive diagnosis of leptospirosis in patients presenting with severe clinical suspicion of the disease.

## 7. Prognosis and Mortality

The severe form of leptospirosis accounts for 10% of all reported cases [12]. Without early recognition and diagnosis, the severe form usually presents with rapid multiple organ dysfunction, including acute renal failure, acute hepatic failure, and acute encephalopathy. In most studies, the mortality is over 10–15% in patients with Weil’s disease and more than 50% in cases of pulmonary hemorrhage [99]. Notably, death is uncommon in patients without AKI. There are some studies that have revealed the risk factors of mortality related to age, mental status at the time of diagnosis, abnormal repolarization characteristics on electrocardiography, and thrombocytopenia [100,101,102]. According to a previous study [103], thrombocytopenia is closely correlated with the occurrence of AKI and is described in all anicteric cases with AKI. Thrombocytopenia with AKI also appears independently of disseminated intravascular coagulation and may be concurrent with severe endotoxemia [104]. Thus, thrombocytopenia may be a sign of an acute form of leptospirosis kidney disease. The conclusion regarding leptospirosis-related CKD is still controversial. AKI may predispose the patient to develop CKD and end-stage renal disease, but a recently published systematic review argued that no definite correlation exists between leptospirosis and CKD [49].

Many studies have explored the role of biomarkers to predict the development of AKI, although so far there is no consensus on the recommendation of using biomarkers in terms of routine clinical practice [105]. One of the most vigorous biomarkers for AKI is neutrophil gelatinase-associated lipocalin (NGAL). Several previous studies have used NGAL as an early marker of AKI and as an outcome predictor [106,107]. Srisawat et al. [107] examined the role of NGAL as an early marker and outcome predictor of leptospirosis-associated AKI in multi-center research involving 113 leptospirosis cases across Thailand. It’s worth noting that AKI developed in 41 of the 113 patients (37 percent). Patients with developing AKI had considerably greater urine and plasma NGAL levels than those without. The area under the receiver operating characteristic (ROC) curve for urine and plasma NGAL levels associated with AKI was 0.91 and 0.92, respectively. In this particular situation, however, neither of them appeared to have a potential role as a predictor of renal recovery. Perhaps, inhomogeneous samples (acute vs. convalescent samples) could trouble the outcomes. Activating transcription factor 3 (ATF3), a transcriptional factor involved in either anti-apoptosis or anti-inflammation process during systemic infection may be an interesting molecule in terms of leptospirosis with AKI [108]. In the sepsis-AKI setting, urinary ATF3 increased at the same days of increased serum creatinine which demonstrated the benefit of urinary AFT3 over urinary NGAL in predicting AKI [109]. Urinary ATF3 did not increase before serum creatinine, urinary ATF3 but not urinary NGAL would be a good additive biomarker for supporting the onset of AKI in sepsis condition with only a subtle increase of serum creatinine, including leptospirosis related AKI.

## 8. Treatments

### 8.1. Specific Treatments for Leptospirosis

The treatment of leptospirosis-related kidney disease usually depends on the clinical symptoms, particularly in the early phase of infection. Therefore, early recognition and diagnosis of leptospirosis kidney disease are the principal factors driving favorable outcomes. In severe leptospirosis cases, the recommended intravenous antibiotics must be promptly prescribed at the time of diagnosis; these include 0.5–1 g ampicillin every 6 h, 1 g ceftriaxone every 12 h, or 1 g cefotaxime every 6 h. Notably, a study from Thailand showed that administration of 1.5 million units of intravenous sodium penicillin G every 6 h is equally effective to ceftriaxone in patients with severe leptospirosis [110]. Once-daily dosing has the added benefit of intramuscular administration in an out-patient setting as an alternative to intravenous administration. However, adult outpatients with an early onset of infection should receive either 100 mg doxycycline twice a day or 500 mg azithromycin daily. Antibiotic treatment is effective within 7–10 days of injection, but the injection of 5 million units/day benzyl penicillin should be prescribed for only 5 days. Patients who are hypersensitive or allergic to the penicillin group may be given 250 mg erythromycin four times a day for 5 days or 100 mg doxycycline twice daily for 10 days. Tetracycline is contraindicated in children, pregnant women, and renal insufficiency patients [111].

### 8.2. Sepsis and Organ Failure in Leptospirosis

Sepsis in leptospirosis, with or without shock, can occur as an unusual presentation, primarily in urban areas [29]. Similar to general sepsis management, the treatment of sepsis in leptospirosis is based on rapid administration of the correct antibiotic and the best supportive care [112]. As such, fluid administration is the cornerstone of sepsis resuscitation. In patients with fluid responder (less than 40% of septic patients), the stroke volume increases by 10–15% after a fluid challenge (250–500 mL), following the Frank–Starling principle (as the preload increases, the stroke volume increases until the optimal preload is achieved) [113]. With the optimal preload, the further fluid administration does not increase the stroke volume but increases arterial pressure, venous pressure, pulmonary hydrostatic pressure, and natriuretic peptide (a fluid shifting inducer from the intravascular portion into the interstitial space). Increased venous pressure (and renal subcapsular pressure) decreases the glomerular filtration rate (GFR) of the kidney. According to the Acute Dialysis Quality Initiative (ADQI), fluid therapy in sepsis divides into rescue (high-volume resuscitation), optimization, stabilization, and de-escalation [114] depending on the individual patient. In the de-escalation phase, a reduction in total fluid administration, diuretics, and/or renal replacement therapy (RRT) might be necessary. For the fluid composition, normal saline (or 0.9% NaCl; a non-physiologic solution) might cause hyperchloremic metabolic acidosis that results in decreased renal blood flow [115]. Synthetic hydroxyethyl starch is potentially nephrotoxic [115]. Although normal saline is currently the main fluid replacement used in sepsis-AKI due to the availability with a reasonable price worldwide, a limited volume of normal saline with partial use of other fluid preparations might be beneficial.

Although acidosis is common in patients with sepsis, bicarbonate treatment is not recommended unless the blood pH is lower than 7.15 because sodium bicarbonate infusion leads to hypernatremia, hypervolemia, intracellular shifting of calcium-induced hypocalcemia, intracellular acidosis, and impaired oxygen delivery [116]. In contrast, the strategies for tissue perfusion improvement (proper respiratory support, and adjusted normal saline volume with other balance solutions) should be considered. Tris-hydroxy-methyl aminomethane (THAM), a weak base with intracellular diffusion that is excreted through the kidneys, is mentioned to reduce intracellular acidosis but causes hyperkalemia, hypoglycemia, pseudo hyponatremia, and an increased osmole gap, especially in patients with pre-existing renal dysfunction [117]. Because the reduced vascular tone is a major cause of hypotension and renal injury in sepsis, norepinephrine restores the normal capillary velocity, filtration pressure, mean arterial pressure, and increases renal medullary circulation without renal blood flow alteration, leading to improved renal function, using as the first-line drug for septic shock.

For the rapid reversal of AKI (due to direct toxins, hypotension and hypovolemia of leptospirosis), topics of renal replacement therapy (RRT); indications, timing, modality, and delivered dose should be applied. Accordingly, the common RRT indications, “A-E-I-O-U”; Acidosis, Electrolyte disturbance, Intoxication, fluid Overload, and Uremia should be used as severe metabolic acidosis, fluid overload, and uremia are the top three RRT indications in leptospirosis. For RRT modality, daily dialysis may provide superior outcomes to alternate-day dialysis in severe leptospirosis (Weil’s syndrome) [118] and extracorporeal blood purification (absorption therapy with polymyxin B or other cytokine absorbents) might be beneficial [119], especially for the hemodynamic improvement [120], but are still inconclusive. Therefore, the proper biomarkers for several aspects (i.e., stress, injury, functional loss, and recovery) for a proper selection of treatment methods are urgently needed. Among them, the base excess (BE) that is lower than −5 might be associated with the success of renal support discontinuation from our experiences (unpublished data). On the other hand, in leptospirosis-related acute liver failure, extracorporeal support systems do not demonstrate any survival advantage in clinical studies and renal support is not recommended in AKI-superimposed chronic liver injury [121]. Nevertheless, renal support may be considered only in patients with reversible causes [122].

## 9. Conclusions

The most common human and veterinary zoonosis is leptospirosis which is expected to cause approximately one million cases per year around the world. Rats, the main reservoir of *Leptospira*, thrive in places with inadequate infrastructure in developing countries. In developed countries, this disease is usually linked to water-related leisure or occupational activities. Humans are usually infected by getting in direct contact with the urine of infected animals, either directly or indirectly. Leptospirosis is frequently misdiagnosed as other acute febrile illnesses due to its nonspecific symptoms. As a result, the prevalence of leptospirosis is likely underreported but has an economic impact due to the reduction of several outputs and causing premature animal death. Numerous factors participate in leptospirosis-related AKI; for example, direct *Leptospira* nephrotoxicity, hyperbilirubinemia, rhabdomyolysis, and sepsis. These factors result in high mortality and morbidity rates. Accordingly, a high suspicion of leptospirosis followed by timely treatment, especially with an appropriate antibiotic along with the best supportive care, could improve complications in severe disease cases.

## Figures and Tables

**Table 1:** Laboratory tests for leptospirosis diagnosis.

Direct Methods	Indirect Methods
Microscopy	Genus-specific antibody tests
DFM, phase contrast	IHA, ELISA, Leptospirosis IgM dipstick, MCAT
Staining	Serovar-specific antibody test
Warthin-Starry silver stain, immunohistochemistry, immunofluorescence, immunoperoxidase	MAT
Isolations of leptospires	
DNA hybridization or DNA probe	
Animal inoculation	
DNA amplification	
PCR, LAMP, NGS, qPCR	

LAMP, loop-mediated isothermal amplification; PCR, polymerase chain reaction; NGS, next-generation sequencing; IHA, indirect hemagglutination; ELISA, enzyme-linked immunosorbent assay; IgM, immunoglobulin M; MAT, macroscopic agglutination test; MCAT, microcapsule agglutination test; DNA, deoxyribonucleic acid; DFM, dark-field microscopy.

## Data Availability

Not applicable.
